# Investigation of potential quality indicators for raw laver (*Pyropia* spp.) standardization: a collaborative approach between traditional assessment and analytical chemistry

**DOI:** 10.3389/fnut.2025.1676911

**Published:** 2025-10-01

**Authors:** Seul-Ki Park, Gyuseok Lee, Gi-Un Seong, Du-Min Jo, Young-Mog Kim, Jeong-Seok Cho, Jeong-Ho Lim, Kee-Jai Park

**Affiliations:** ^1^Smart Food Manufacturing Research Group, Korea Food Research Institute, Wanju, Republic of Korea; ^2^National Marine Biodiversity Institute of Korea (Mabik), Seochun, Republic of Korea; ^3^Department of Food Science and Technology, Pukyong National University, Busan, Republic of Korea; ^4^Research Center for Marine Integrated Bionics Technology, Pukyong National University, Busan, Republic of Korea; ^5^Food Convergence Research Division, Korea Food Research Institute, Wanju, Republic of Korea

**Keywords:** quality indicator, raw laver, *Pyropia* spp., seafood, standardization, multivariate analysis

## Abstract

**Introduction:**

Raw laver (*Pyropia* spp.) quality assessment is largely subjective and lacks scientific standardization.

**Methods:**

This study established objective quality markers by integrating biochemical profiling with market-based valuation. Twenty-two samples from Seocheon, South Korea (Jan -Mar 2024) were classified into high, medium, and low quality based on auction price and total free amino acid (TFAA) content. Proximate composition and amino acid profiles were determined using AOAC methods and HPLC. Multivariate models (PCA, PLS-DA, OPLS-DA) were applied to identify key discriminants.

**Results:**

The OPLS-DA model achieved excellent performance (R^2^X = 0.790, R^2^Y = 0.916, Q^2^ = 0.911) and clear group separation. Alanine, glutamic acid, and aspartic acid were critical markers, with alanine showing the strongest correlation with TFAA (r = 0.93). The PLS-DA model achieved 100% classification accuracy in both training and test sets.

**Discussion:**

These findings provide a robust scientific basis for raw laver quality grading, supporting transparent market practices and industrial standardization.

## Highlights

- Amino acid profiles were identified as key indicators of raw laver quality.
- Established TFAA content is a robust marker for grading raw laver.
- PCA, PLS-DA, and OPLS-DA enable precise classification of laver quality.
- 100% classification accuracy in raw laver quality was obtained.
- The TFAA-based grading system was validated for industrial quality control.

## 1 Introduction

The red algae *Pyropia* spp. (*Rhodophyta*), traditionally consumed in East Asia, have gained global attention as a nutrient-rich “superfood” owing to their high protein content (25–50%), dietary fiber (30–40%), essential minerals (iodine, iron, calcium), and vitamins (A, B12, C) (1, 2). Free amino acids (FAAs), such as glutamic acid and alanine, contribute significantly to the umami flavor profile and nutritional value of laver, while phycocyanin and other antioxidants enhance its status as a functional food (3–7). South Korea, a major global producer, is projected to harvest approximately 350,000 metric tons of *Pyropia* in 2023, with exports exceeding USD 800 million (8). Seocheon-gun in Chungcheongnam-do, South Korea, accounts for 93–95% of the laver production of the province, capitalizing on its pristine coastal waters. Despite this economic importance, raw lever quality is still judged by subjective sensorial evaluations during auctions, and dried laver is graded visually based on attributes such as color, thickness, and foreign matter (9). These subjective methods can reduce consumer trust and hinder transparency in the seafood industry. Recent advances in algal research have promoted the use of objective quality assessment tools. Non-destructive technologies, such as near-infrared spectroscopy, attenuated total reflectance Fourier-transform infrared (ATR-FTIR) spectroscopy, and hyperspectral imaging combined with multivariate analysis, have enabled rapid quality evaluation of dried laver, particularly for moisture, protein, and texture parameters (9–12). For instance, hyperspectral imaging integrated with partial least squares discriminant analysis (PLS-DA) has demonstrated high accuracy in grading dried laver by predicting key quality parameters (13). Similarly, ATR-FTIR coupled with principal component analysis (PCA) and PLS regression (PLSR) has identified metabolomic markers associated with commercial quality (10). However, these approaches have focused exclusively on dried laver and largely ignored raw laver. Although dried laver is the primary form assessed and traded in the market, its quality ultimately derives from the biochemical attributes of raw laver. Quality evaluation studies on raw laver remain scarce, largely due to the practical difficulties in sample acquisition and handling. Without a clear understanding of the intrinsic biochemical composition of raw laver and the environmental factors influencing it, it is challenging to elucidate the fundamental causes underlying the quality variation observed in dried products. Therefore, the present study provides a crucial first step in profiling the nutritional and biochemical indicators of raw laver, which may subsequently inform the grading and standardization of dried laver.

Raw laver (*Pyropia* spp.), which is auctioned before drying and processing, still lacks scientifically validated quality indicators. Current industrial practices often involve blending raw laver from different regions to enhance final product quality; however, these blends are based on empirical sensory evaluations rather than quantitative biochemical data. Unlike fish, which are classified by size or weight, the filamentous structure and phenotypic plasticity of laver complicate objective classification. Thus, identifying measurable biomarkers for raw laver quality remains a key challenge with broad implications for aquaculture efficiency and market fairness. This study addresses this gap by correlating the biochemical composition of raw *Pyropia* spp. from Seocheon with commercial auction values. A total of 22 raw laver samples, stratified by auction prices from January to March 2024, underwent proximate composition analysis (crude protein, lipids, and ash) and amino acid profiling using high-performance liquid chromatography (HPLC). Multivariate models (PCA, PLSR) were used to identify key discriminant biomarkers predictive of auction-based quality. By integrating market prices with metabolomic profiles, this study offers a transparent, science-based framework for assessing raw laver quality and enhancing trust throughout the supply chain.

## 2 Materials and methods

### 2.1 Sample preparation and value assessment

Raw laver (*Pyropia* spp.) samples were collected from the Songsuk Port auction market in Seocheon, Chungcheongnam-do, South Korea, with assistance from the local Fisheries Cooperative Association, since raw laver is not directly accessible to consumers. Samples were obtained biweekly from January to March 2024, resulting in seven collection events and a total of 22 distinct samples (Table 1; Supplementary Table 1). Immediately after collection, samples were stored in polystyrene containers and transported to the laboratory at temperatures below 4 °C. Upon arrival, a portion of each sample was immediately frozen and subsequently freeze-dried at −70 °C to obtain powdered material suitable for long-term storage and subsequent analyses of amino acid content and proximate composition. Auction prices for raw laver are determined by auctioneers based on multiple factors, including quality, volume, and market supply. To evaluate the relationship between composition and commercial value, detailed information on auctioned quantity, final sale price, and relevant metadata was recorded for each sample and summarized in Table 1 and Supplementary Table 1. Additionally, the Seocheon auction market primarily handles laver harvested from nearby aquaculture sites, as large tidal ranges in this region restrict vessel access from more distant areas. The harvest season in Seocheon typically commences in mid-December and concludes by late March, which is shorter than in southern coastal areas, where harvesting can extend until mid-April. Our sampling, conducted from January to March 2025, encompassed more than 70% of the harvesting period in the Seocheon region. The geographic distribution of the main aquaculture farms supplying this auction market is presented in the Supplementary Figure 1.

**Table 1:** List of raw laver (*Pyropia* spp.) samples collected from the Seocheon region between January and March 2024, including production quantity, auction price, grade category, and calculated base price.

Date	Label	Quantity^*^	Price (USD, 120 kg)^*^	Category^**^	Calculated price (USD, $) by developed equation		
					Unit price (P_i_/kg)	Base price (P_ij_/kg)	Price balance ($)
January 30, 2024	J1H1	80	$183.00	High	1.52	1.45	+0.07
	J1H2	71	$173.23	High	1.44		−0.01
	J1L1	50	$146.92	Low	1.22		−0.23
February 6, 2024	F1H1	70	$151.46	High	1.26	1.15	+0.12
	F1L1	60	$119.62	Low	1.00		−0.15
February 13, 2024	F2H1	89	$137.31	High	1.14	1.04	+0.11
	F2L1	70	$73.23	Low	0.61		−0.43
February 20, 2024	F3H1	55	$160.15	High	1.33	1.26	+0.07
	F3H2	15	$165.38	High	1.38		+0.12
	F3L1	30	$140.69	Low	1.17		−0.09
	F3L2	80	$145.54	Low	1.21		−0.05
February 26, 2024	F4H1	70	$186.15	High	1.55	1.53	+0.02
	F4H2	74	$205.31	High	1.71		+0.18
	F4L1	29	$78.46	Low	0.65		−0.88
	F4L2	36	$163.85	Low	1.37		−0.17
March 6, 2024	M1H1	75	$193.08	High	1.61	1.69	−0.08
	M1H2	60	$210.23	High	1.75		+0.06
	M1L1	70	$146.08	Low	1.22		−0.47
	M1L2	27	$153.08	Low	1.28		−0.42
March 13, 2024	M2L1	65	$216.23	Low	1.80	1.85	−0.04
	M2L2	40	$211.62	Low	1.76		−0.08
	M2L3	51	$212.38	Low	1.77		−0.08

^*^Quantity refers to one package (120 kg). All prices in this table were converted from Korean Won (KRW) to US Dollars (USD) at an exchange rate of 1 USD = 1,300 KRW. ^**^The samples were categorized as “high” or “low” based on the raw laver price on the auction day when each sample was collected. Detailed information on the auction prices for raw laver on each sampling date is provided in Supplementary Table 1.

### 2.2 Re-categorization of raw laver auction prices

The auction price of laver is traditionally determined by market conditions, with quality playing a significant role in price formation. To establish a meaningful correlation between auction prices (as a proxy for perceived quality) and compositional data, we needed to standardize the prices into a normalized “base price.” This approach mitigates the influence of daily market fluctuations and enables consistent comparisons across different collection dates. The base price was calculated using four key parameters: total daily harvest volume, total auction revenue, daily average unit price, and the unit price of the collected sample. These values were derived using the following equations:

For the statistical analysis of raw laver auction prices, key variables were calculated using the harvest quantity (W_ij_, in bags) and unit price (P_ij_, in KRW/bag) from fishing ground j on day i. The total daily sales (P_total,i_, KRW) were calculated using Equation 1:


(1) $$P_{\text{total},i}=\sum_{j}W_{\mathrm{ij}}\times P_{\mathrm{ij}}$$

The total daily harvest volume (W_total,i_, bags) was determined as the sum of all fishing grounds, as shown in Equation 2.


(2) $$W_{\text{total},i}=\sum_{j}W_{\mathrm{ij}}$$

The daily average unit price (P_ave,i_, KRW/bag), which was used as a reference point for base price calculations, was computed by dividing the total revenue by the total harvest volume, as expressed in Equation 3.


(3) $$P_{\mathrm{ave},i}=\frac{P_{\text{total},i}}{W_{\text{total},i}}=\frac{\sum_{j}W_{\mathrm{ij}}P_{\mathrm{ij}}}{\sum_{j}W_{\mathrm{ij}}}$$

Finally, the difference between a fishing ground’s unit price and the daily average price (ΔP_ij_, KRW/bag) was calculated using Equation 4, indicating a price premium or discount, associated with quality variations:


(4) $$\Delta P_{\mathrm{ij}}=P_{\mathrm{ij}}-P_{\mathrm{ave},i}$$

where i represents the auction date, j denotes the fishing ground number, W_ij_ is the number of bags harvested from fishing ground j on day i (bags), and P_ij_ is the corresponding unit price (KRW/bag). In this analysis, one standard bag of raw laver was defined as having 120 kg.

### 2.3 Proximate composition analysis

The proximate composition of raw laver, including moisture, ash, crude fat, and crude protein, was determined following the official methods of the AOAC (14). Moisture content was measured using the atmospheric oven-drying method. Approximately 5 g of homogenized sample was dried at 105 °C until a constant weight was achieved (typically over 5 h). Ash content was determined by incinerating approximately 5 g of the sample in a pre-weighed porcelain crucible. Samples were pre-carbonized and then turned into ash in a muffle furnace at 550 °C for at least 12 h until a whitish-gray ash was obtained. Crucibles were cooled to 200 °C, transferred to a desiccator, and weighed at 25 ± 0.5 °C to ensure that the weight was kept constant. Crude protein was quantified using the Kjeldahl method by using an automatic protein analyzer (Kjeltec™ 8,400 Analyzer Unit; FOSS Tecator AB, Höganäs, Sweden). Total nitrogen content was measured and multiplied by a conversion factor of 6.25 to calculate crude protein content. Crude fat was analyzed using Soxhlet extraction. About 1 g of freeze-dried sample was placed in a cellulose thimble and extracted with petroleum ether using an automatic Soxhlet extractor (SER 158 Series; Velp Scientifica, Usmate, Italy).

### 2.4 Amino acid analysis

#### 2.4.1 Sample preparation

Two distinct extraction protocols were applied to quantify free amino acids (FAAs) and constituent amino acids (CAAs) in raw laver samples. For FAA extraction, freeze-dried raw laver (0.5 g) was placed in a 50 mL conical tube and extracted sequentially using 70% ethanol. Each extraction involved stirring at room temperature for 1 h, followed by centrifugation at 10,000 × g for 10 min. Supernatants were collected via filtration into a flat-bottom flask. A second extraction was performed by adding 25 mL of 70% ethanol to the residue and this process was repeated. The combined extracts were concentrated using a rotary evaporator, then resuspended in 2 mL of 0.1 N HCl. The solution was split into two 1 mL aliquots (E-tubes) and filtered using a 0.2 μm filter. For HPLC analysis, 100 μL of the filtered extract was diluted with 900 μL of filtered 0.1 N HCl, shaken for 10 min, and 500 μL was transferred into an HPLC vial. For CAA extraction, freeze-dried raw laver (0.5 g) was hydrolyzed with 4 mL of 6 N HCl in a sealed tube at 110 °C for 24 h. After hydrolysis and cooling, the hydrolysate was vortexed and 1 mL was transferred to an E-tube and centrifuged at 10,000 × g for 10 min. From the supernatant, 50 μL was transferred to each of two E-tubes containing 950 μL of borate buffer (pH 10.2), followed by vortexing. The mixtures were then centrifuged at 10,000 × g for 10 min, and the resulting supernatants were filtered using a 0.2 μm filter into a 15 mL conical tube. For HPLC analysis, 300 μL of borate buffer was mixed with 300 μL of the filtered hydrolysate, vortexed, and 500 μL of the mixture was transferred to an HPLC vial (15).

#### 2.4.2 HPLC system and detection

Amino acid analysis was performed using an Agilent 1,260 Infinity II HPLC System (Agilent Technologies, Santa Clara, CA, United States) equipped with a diode array detector (DAD). To enhance sensitivity and resolution, automated pre-column derivatization was employed for both FFAs and CAAs. Primary amino acids were derivatized using o-phthalaldehyde (OPA), while secondary amino acids were derivatized using a combination of o-phthalaldehyde and 9-fluorenylmethyl chloroformate (FMOC).

#### 2.4.3 Chromatographic conditions

Chromatographic separation was achieved using an AdvanceBio AAA column (4.6 × 100 mm, 2.7 μm; Agilent Technologies, Santa Clara, CA, United States) maintained at 40 °C. The mobile phase consisted of: solvent A is HPLC water (Sigma-Aldrich, St. Louis, MO, United States), solvent B (40 mM sodium phosphate (dibasic) containing 0.1% phosphoric acid), and solvent C (methanol:acetonitrile:water; 45:45:10, v/v/v). Elution was performed at a flow rate of 0.8 mL/min under the following gradient program: 0–0.35 min, 98% B, 2% C; 0.35–13.40 min, linear gradient to 43% B, 57% C; 13.40–13.50 min, linear gradient to 0% B, 90% C, 10% A; 13.50–16.70 min, 0% B, 90% C, 10% A; 16.70–16.80 min, linear return to initial conditions 98% B, 2% C; 16.80–19.00 min, 98% B, 2% C. Detection was performed using a DAD at 338 nm (bandwidth 10 nm), with a reference wavelength set at 390 nm (bandwidth 20 nm).

#### 2.4.4 Automated pre-column derivatization

Derivatization was carried out using an automated protocol by using Agilent 1,260 Infinity II Multisampler (Agilent Technologies). The reagents included: borate buffer (0.4 N, pH 10.2), OPA reagent (10 mM OPA in borate buffer with 3-mercaptopropionic acid), and FMOC reagent (2.5 mM in acetonitrile). The derivatization sequence followed a previously described procedure: 2.5 μL of borate buffer and 1.0 μL of sample were mixed, to which 0.5 μL of OPA reagent was added, mixed in, and allowed to react for 1.0 min. Then, 0.4 μL of FMOC reagent was mixed in and allowed to react for 0.5 min, followed by injection into the HPLC system (16).

#### 2.4.5 Data analysis

Amino acids were identified by comparing sample retention times with those of known standard amino acids. Quantification was performed using calibration curves constructed from standard amino acid mixtures (Agilent Technologies, part no. 5062-2,478) at concentrations ranging from 1 to 100 pmol/L. Results for FFAs and CAAs were expressed as mg of amino acid per 100 g of sample (dry weight basis).

### 2.5 Data processing for multivariate statistical analysis

Multivariate statistical analyses were performed using SIMCA version 18.0.1 (Umetrics, Umeå, Sweden). Prior to analysis, raw data were preprocessed using unit variance (UV) scaling to normalize the influence of variable magnitude (17). PCA was first employed to assess inherent data variability and detect potential outliers. PCA extracts orthogonal principal components (PCs) that maximize variance in the data by forming linear combinations of variables (18). Score and loading plots were used to interpret sample clusters and identify key contributing variables. Partial Least Squares (PLS) regression was applied to explore the relationship between independent variables (X: biochemical profiles) and dependent variables (Y: auction prices). PLS projects both X and Y into a latent variable (LV) space, maximizing their covariance (19). Model performance was evaluated using the goodness of fit (R^2^) and predictive ability (Q^2^), calculated via seven-fold cross-validation. Robustness was further validated via permutation testing (200 permutations), with model significance confirmed when the original Q^2^ exceeded all permuted Q^2^ values (20). Variables with variable importance in projection (VIP) scores > 1.0 were considered significant contributors to the model (21). For classification tasks (e.g., high- vs. low-price laver), orthogonal PLS discriminant analysis (OPLS-DA) was employed. OPLS-DA improves model interpretability by separating variation in X into predictive (related to Y) and orthogonal (unrelated to Y) components (22). Model validity was confirmed using CV-analysis of variance (ANOVA) (*p* < 0.05) and permutation tests. Thresholds of R^2^Y < 0.4 and Q^2^ < 0.05 were used to flag overfitting (23). All models were constructed using mean-centered data and were validated according to established chemometric best practices (17).

### 2.6 Model development and performance evaluation—confusion matrix

Partial least squares discriminant analysis (PLS-DA) is a supervised classification technique that integrates the dimensionality reduction of PLS with class discrimination functionality. It projects high-dimensional input variables onto a lower-dimensional LV space, maximizing the covariance between predictors and categorical response variables. These latent variables are then used in a linear decision function to classify samples into predefined groups. PLS-DA is particularly effective for datasets with multicollinearity and a high predictor-to-observation ratio (24). To evaluate classification performance, a confusion matrix was constructed by comparing actual class labels with predicted labels. This matrix summarizes the number of correct and incorrect predictions across all classes, enabling calculation of key performance metrics: accuracy, sensitivity (recall), specificity, and precision. These metrics offer a comprehensive view of the model’s classification ability, particularly in distinguishing between true and false positives, and true and false negatives. Model development and evaluation were conducted using a custom Python script. All analyses were performed using Python 3.10.16, and the scikit-learn library (version 1.6.1) was used for implementing the PLS-DA algorithm and computing the confusion matrix and associated performance metrics.

### 2.7 Statistical analysis

All experiments were conducted using five biological replicates, and results are presented as mean ± standard deviation (SD). Statistical comparisons between groups were conducted using one-way ANOVA, followed by Duncan’s multiple range test for *post-hoc* analysis at a significance level of *p* < 0.05, using SPSS 29 (IBM Corp., Armonk, NY, United States) (25, 26). Before applying ANOVA, assumptions of normality and homogeneity of variance were verified using the Shapiro–Wilk test and Levene’s test, respectively. Multivariate pattern recognition, PCA, and PLSR were performed using SIMCA 18.0.1 (Umetrics). Raw data were preprocessed using mean-centering and UV scaling to mitigate biases from differing variable units (17). PCA was used to explore internal data structures, identify clusters, and detect outliers. PLSR was employed to model relationships between the biochemical profile (X-matrix) and auction price (Y-matrix). Model validity was evaluated using seven-fold cross-validation, with performance assessed using predictive ability (Q^2^) and goodness of fit (R^2^). Variables with VIP scores > 1.0 were considered significant contributors to the model (21).

## 3 Results and discussion

### 3.1 Proximate composition of raw laver

Raw laver samples collected from Seocheon between January and March 2024 are summarized in Table 1. Proximate composition analysis revealed the following ranges: a moisture content of 86.84–90.82%, a crude protein content of 1.23–4.61 g/100 g, a crude lipid content of 0.040–0.070 g/100 g, and an ash content of 2.03–3.94% (Table 2). Moisture content showed significant seasonal variation, with the highest value (90.82% ± 0.22%) observed in sample F3H1 (February 20) and the lowest (86.84% ± 0.38%) in sample J1H1 (January 30). These results align with Mok et al. (27), who reported moisture levels of 89.9% ± 1.4% in raw laver (*Pyropia yezoensis*) from Korean coastal waters, reflecting physiological changes across the harvest season. Crude protein content was relatively higher in January (3.70–4.61 g/100 g) and decreased by March, reaching a minimum of 1.23 ± 0.07 g/100 g in sample F4L2 (February 26). This pattern supports earlier findings that total amino acid content in *Pyropia* species declines from November to March (27). Crude lipid levels remained consistently low across all samples (0.040–0.070 g/100 g), consistent with Dawczynski et al. (28), who reported lipid contents of around 1% in edible seaweeds. The lowest lipid content (0.040 ± 0.015 g/100 g) was recorded in the January sample J1H1. Ash content peaked at 3.94% ± 0.59% in sample F4L1 (February 26) and dropped to a minimum of 2.03% ± 0.12% in sample F2H1 (February 13). When converted to dry weight (15–30%), these values are consistent with ash contents reported in Seocheon laver (29). January samples (J1H1, J1H2, and J1L1) showed relatively high ash levels (3.40–3.66%), which decreased mid-February (2.03–2.57%) and increased again in late February and March. These fluctuations likely reflect seasonal shifts in seawater mineral content and the physiological state of the laver (29, 30). The seasonal decline in crude protein content correlated with developmental stages and environmental factors. On a dry weight basis (10–35%), the protein content fell within the expected range for *Porphyra* species, such as Portuguese samples (23.7%) (31). Similarly, the ash content (15–30% dry weight) was lower than that of Canadian Porphyra spp. (12.23–26.30%) (32) but comparable to that of Portuguese samples (19.07%). These differences may be attributed to regional variations in seawater chemistry, environmental conditions, and species-specific traits. Environmental factors have a pronounced impact on the proximate composition and safety profile of raw laver. Park et al. (33) reported that crude protein content decreases significantly with increasing sea surface temperature (VIP = 2.00, r = −0.695, *p* < 0.05), while cadmium accumulation increases as salinity decreases (VIP = 1.69, r = −0.667, *p* < 0.05). These findings align with the observed seasonal fluctuations in moisture, crude protein, lipid, and ash contents in samples collected from Seocheon between January and March 2024. Seasonal declines in protein and shifts in ash content likely reflect both physiological changes and environmental variability, including seawater temperature and salinity (33). Integrating real-time monitoring of these environmental factors with biochemical analysis provides a robust framework for optimizing harvest timing and improving quality control, supporting sustainable production in the seaweed aquaculture industry.

**Table 2:** Proximate composition of raw laver (*Pyropia* spp.) samples from Seocheon, detailing moisture, ash, crude fat, and crude protein contents.

Date	Label	Proximate composition (g/100 g)			
		Moisture contents	Crude protein	Crude lipid	Ash
January 30, 2024	J1H1	86.84 ± 0.38^h*^	4.50 ± 0.18^a^	0.040 ± 0.015ᵇ	3.66 ± 0.08ᵃᵇ
	J1H2	87.5 ± 0.61^efgh^	3.70 ± 0.50^b^	0.070 ± 0.013ᵃ	3.40 ± 0.30^bcd^
	J1L1	87.42 ± 0.3^fgh^	4.61 ± 0.53^a^	0.070 ± 0.005ᵃ	3.57 ± 0.15^abc^
February 6, 2024	F1H1	88.92 ± 0.47^cd^	3.53 ± 0.50^bc^	0.060 ± 0.015ᵃᵇ	3.41 ± 0.18^bcd^
	F1L1	90.13 ± 0.36^ab^	3.41 ± 0.09^bcd^	0.060 ± 0.011ᵃᵇ	3.53 ± 0.17ᵃᵇᶜᵈ
February 13, 2024	F2H1	88.15 ± 0.36^def^	2.57 ± 0.08^efgh^	0.070 ± 0.015ᵃ	2.03 ± 0.12^g^
	F2L1	87.95 ± 0.31^efg^	2.03 ± 0.12^hi^	0.060 ± 0.016ᵃᵇ	2.57 ± 0.07^f^
February 20, 2024	F3H1	90.82 ± 0.22^a^	2.52 ± 0.07^fgh^	0.050 ± 0.008ᵃᵇ	2.69 ± 0.11ᵉᶠ
	F3H2	90.42 ± 0.30^ab^	2.66 ± 0.06^efg^	0.050 ± 0.008ᵃᵇ	2.75 ± 0.11ᵉᶠ
	F3L1	89.56 ± 0.43^bc^	2.32 ± 0.36^ghi^	0.060 ± 0.017ᵃᵇ	3.35 ± 0.24ᵇᶜᵈ
	F3L2	88.22 ± 0.48^def^	1.99 ± 0.09^hi^	0.070 ± 0.006ᵃ	3.53 ± 0.13ᵃᵇᶜᵈ
February 26, 2024	F4H1	88.95 ± 0.70^cd^	2.97 ± 0.79^cdef^	0.070 ± 0.013ᵃ	3.42 ± 0.19^bcd^
	F4H2	88.40 ± 0.71^de^	3.72 ± 0.22^b^	0.070 ± 0.011ᵃ	3.47 ± 0.17ᵇᶜᵈ
	F4L1	88.06 ± 0.70^defg^	1.75 ± 0.53^i^	0.060 ± 0.021ᵃᵇ	3.94 ± 0.59^a^
	F4L2	88.11 ± 0.53^defg^	1.23 ± 0.07^j^	0.050 ± 0.007ᵃᵇ	3.53 ± 0.59^a^ᵇᶜᵈ
March 6, 2024	M1H1	87.15 ± 0.36^gh^	3.10 ± 0.12^cde^	0.070 ± 0.014ᵃ	3.16 ± 0.18ᶜᵈ
	M1H2	86.97 ± 0.24^h^	3.11 ± 0.11^cde^	0.070 ± 0.015ᵃ	3.08 ± 0.15ᵈᵉ
	M1L1	87.63 ± 0.88^efgh^	2.92 ± 0.29^ef^	0.070 ± 0.014ᵃ	3.27 ± 0.18ᵇᶜᵈ
	M1L2	87.27 ± 0.52^fgh^	3.11 ± 0.11^cde^	0.070 ± 0.012ᵃ	3.29 ± 0.16ᵇᶜᵈ
March 13, 2024	M2L1	87.96 ± 0.37^efg^	2.15 ± 0.12^ghi^	0.060 ± 0.011ᵃᵇ	3.35 ± 0.16ᵇᶜᵈ
	M2L2	87.25 ± 0.44^fgh^	2.23 ± 0.12^ghi^	0.060 ± 0.008ᵃᵇ	3.40 ± 0.09ᵇᶜᵈ
	M2L3	88.10 ± 0.64^defg^	2.04 ± 0.09^hi^	0.060 ± 0.008ᵃᵇ	3.19 ± 0.17ᶜᵈ

^*^Values are shown as mean ± standard deviation (SD). Different superscript letters within a column indicate significant differences according to Duncan’s multiple range test (*p* < 0.05).

In the auction price-based classification (High vs. Low), crude protein content was significantly higher in the high-price group across all sampling dates (*p* < 0.05), suggesting that traditional quality grading indirectly reflects protein content. Conversely, ash content exhibited the opposite trend, with significantly lower values observed in the high-price group. These findings indicate that traditional sensory-based auction pricing aligns well with biochemical data, supporting its continued use as a valid proxy for quality assessment.

### 3.2 Multivariate statistical analysis for grading raw laver

**Figure 1:**
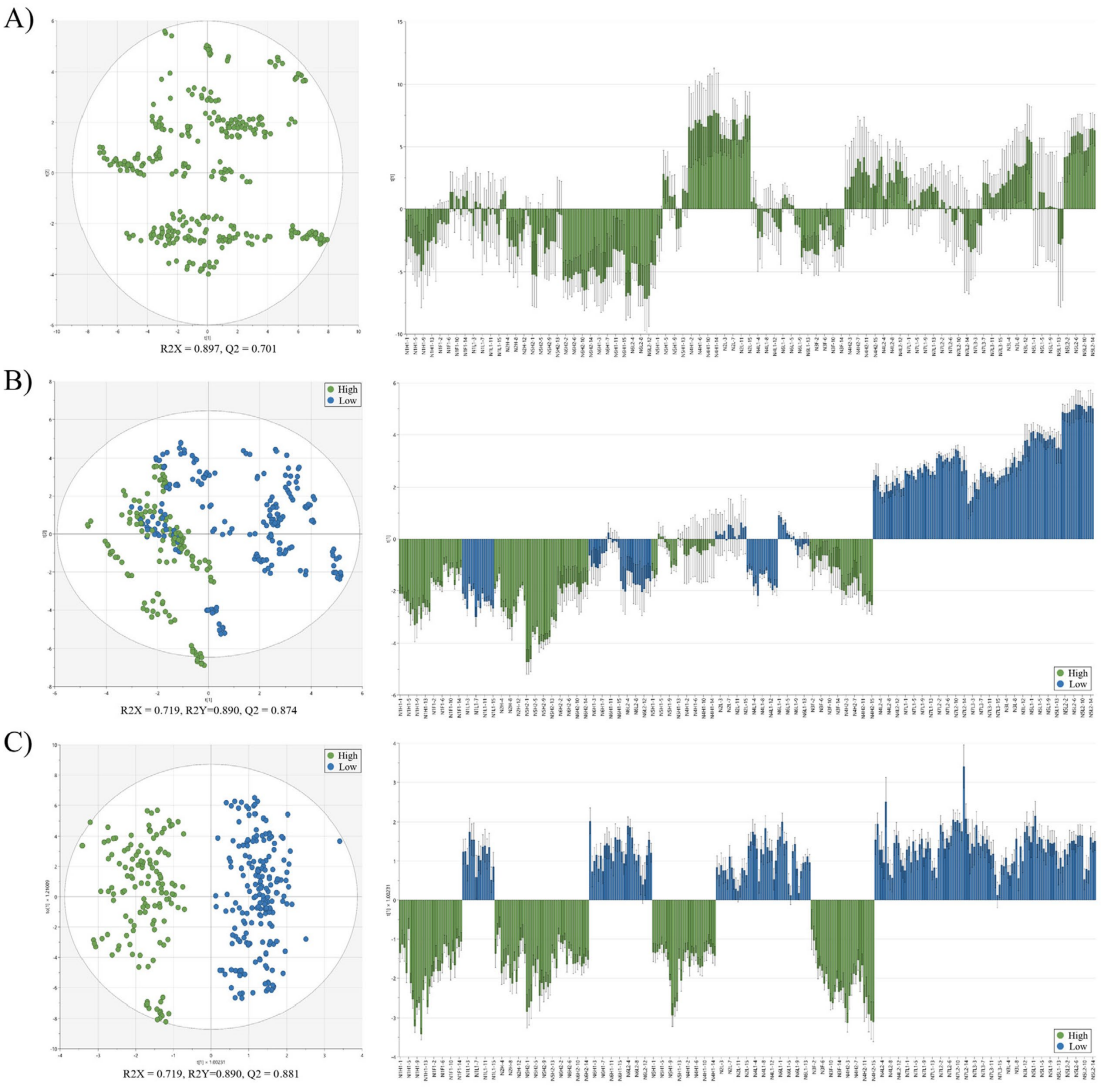
Multivariate analysis of proximate composition and amino acid profiles in raw laver (*Pyropia* spp.) samples collected from Seocheon between January and March 2024. **(A)** Principal component analysis (PCA) score plot and loading plot; **(B)** partial least squares discriminant analysis (PLS-DA) score plot and loading plot; **(C)** orthogonal partial least squares discriminant analysis (OPLS-DA) score plot and loading plot. High and low groups are indicated by different colors. R2X, R2Y, and Q2 values represent model fit and predictive ability.

Classification models were developed to evaluate the ability of compositional profiles, proximate components, and free/CAAs to differentiate quality grades of raw laver based on auction price. PCA, partial least squares discriminant analysis (PLS-DA), and orthogonal partial least squares discriminant analysis (OPLS-DA) were applied to the full dataset, which included proximate composition data and free/CAAs profiles for all collected raw laver samples. Classification was based on auction price, with samples grouped into High and Low categories (Figure 1). In the PCA model (Figure 1A), no group labels were applied. The first (PC1) and second principal components (PC2) together explained 89.7% of the total variance (R^2^X = 0.897). Despite this high variance capture, no distinct clustering patterns were observed between the high- and low-price groups. This finding contrasts with Son and Lee (34), who reported three distinct clusters when analyzing the amino acid compositions of five Korean seaweed species. The lack of separation in our PCA model likely reflects the complexity of the dataset, which spanned multiple collection periods and included both general components and free/constitutive amino acid variables, demonstrating the limitations of PCA for clear pattern recognition for this application. In contrast, the PLS-DA model (Figure 1B), which incorporated auction price-based group labels, demonstrated strong classification performance, with R^2^X = 0.719, R^2^Y = 0.890, and Q^2^ = 0.874. Although some overlap remained between the two groups, partial separation was evident. The high Q^2^ value (> 0.5) indicated strong predictive ability, supporting the effectiveness of the model. The OPLS-DA model (Figure 1C), a refined version of PLS-DA that separates predictive from orthogonal variance for improved interpretability (7), further enhanced classification clarity. The model achieved R^2^X = 0.719, R^2^Y = 0.890, and Q^2^ = 0.881. The score plot revealed clear separation between the high- and low-price groups, along the predictive component (y-axis), while the corresponding loading plot confirmed this separation (Figure 1C). All multivariate statistical models demonstrated strong discriminative performance. Notably, the PLS-DA and OPLS-DA models achieved Q^2^ values exceeding 0.87, indicating excellent predictive capability and practical applicability for quality classification. These results align with the findings by Ye et al. (7), who reported similar OPLS-DA performance in their NMR-based metabolomic analysis of nutritional changes in processed *P. yezoensis*. The effectiveness of such multivariate techniques is further supported by Guo et al. (35), who used LC–MS-based profiling to differentiate arsenic-exposed seaweed metabolomes. Importantly, both PLS-DA and OPLS-DA models establish a meaningful connection between traditional sensory-based quality assessments, as reflected in auction prices, and quantitative compositional data. Specifically, OPLS-DA confirmed a high probability of accurately classifying raw laver samples into high- and low-quality groups.

### 3.3 Multivariate discriminant analysis of laver reclassified

**Figure 2:**
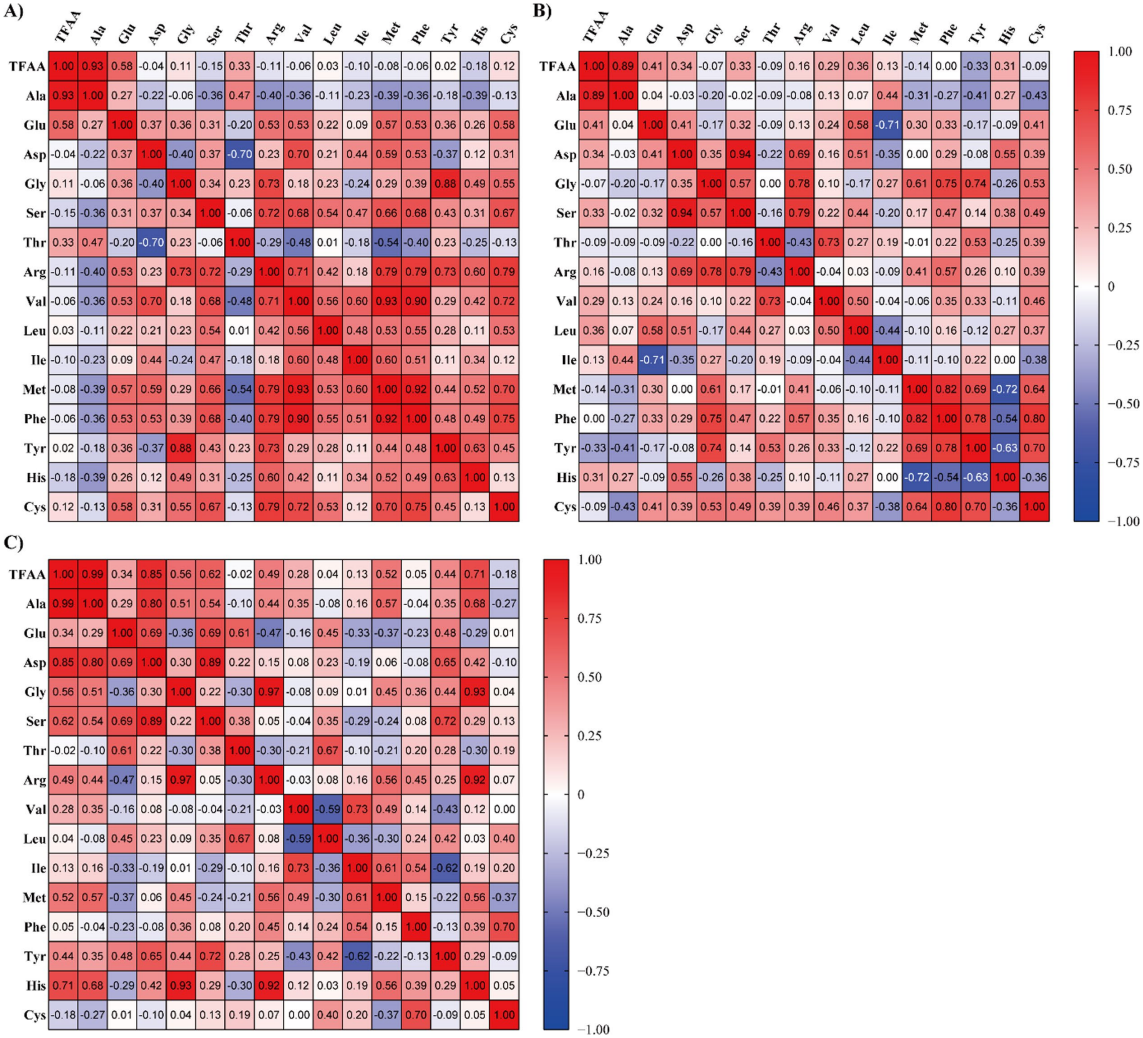
Heatmap visualization of Pearson correlation coefficients among free amino acids in raw laver (*Pyropia* spp.) samples collected from Seocheon (January–March 2024), classified into three quality groups based on total free amino acid (TFAA) content: **(A)** high, **(B)** medium, and **(C)** low. ^*^Color intensity represents the strength and direction of pairwise correlations. Positive correlations are shown in red, and negative correlations in blue.

**Figure 3:**
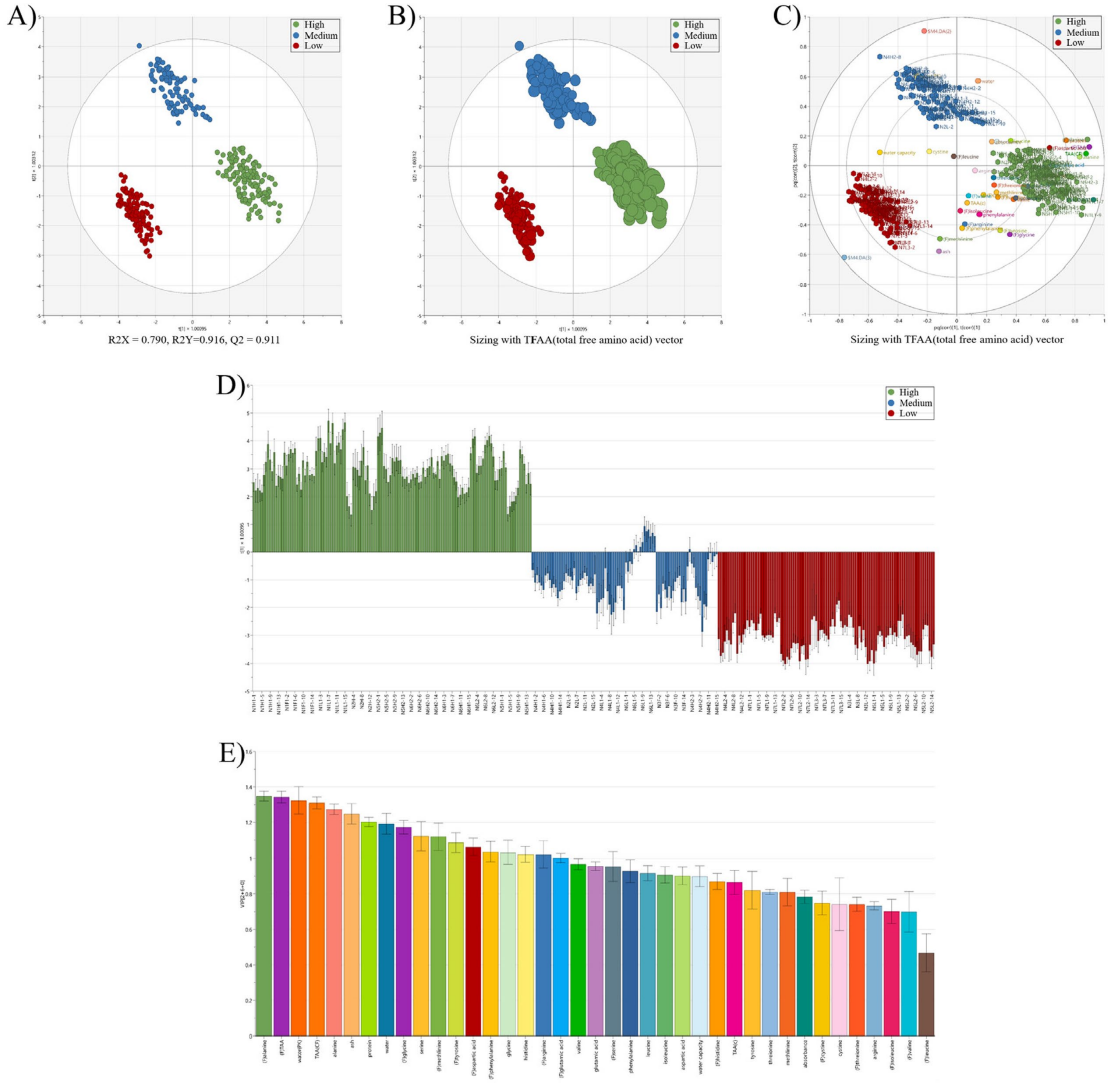
Multivariate discriminant analysis of raw laver (*Pyropia* spp.) samples reclassified (3 groups, high; medium; low) based on total free amino acid (TFAA) content. **(A)** Partial least squares discriminant analysis (OPLS-DA) score plot showing separation of high, medium, and low groups (R2X = 0.700, R2Y = 0.916, Q2 = 0.911); **(B)** OPLS-DA score plot with TFAA vector sizing to visualize the influence of total free amino acid content on sample classification; **(C)** OPLS-DA biplot combining score and loading vectors, illustrating the correlation between samples [green, blue, red circles (●)] and variables (amino acids/nutritional components (●), colored circles); **(D)** loading contribution plot showing variable contributions for discriminating high, medium, and low TFAA groups; **(E)** variable importance in projection (VIP) scores ranking the most significant amino acids contributing to group classification. ^*^Sample groups are color-coded: green (high), blue (medium), and red (low). Model validation parameters R2X, R2Y, and Q2 indicate model reliability and predictive capability. ^*^(C) Nutrient components (e.g., glutamic acid, alanine, aspartic acid) clustered near the high-TFAA group (green) indicate their significant contribution to the group’s distinct profile, suggesting higher abundance and diversity of these compounds in high-TFAA groups samples.

Raw laver (*Pyropia* spp.) samples were reclassified into three quality groups (High, Medium, and Low) based on total free amino acid (TFAA) content (Table 3). In Table 3, the three groups were classified according to TFAA content, with the High group comprising samples with TFAA levels ranging from 129.56 to 167.10 mg/100 g. The Medium group included samples with TFAA contents between 97.91 and 112.30 mg/100 g, while the Low group ranged from 41.09 to 93.02 mg/100 g. Raw laver samples containing 120 mg/100 g or more of TFAA were designated as the High group, those with TFAA levels from 97 to less than 120 mg/100 g as the Medium group, and those with 97 mg/100 g or less as the Low group (Table 3). A heatmap based on Pearson correlation coefficients for each analyzed amino acid is shown in Figure 2, and the results of the multivariate discriminant analysis for the three reclassified groups are shown in Figure 3.

**Table 3:** Three-tier grading system of raw laver (*Pyropia* spp.) samples from Seocheon (January–March 2024), reclassified by total free amino acid (TFAA) content, along with corresponding market price balance information.

New class	Price balance ($)	Label	TFAA (mg/100 g)
High	+0.07	J1H1	158.06 ± 5.12^b^
	−0.01	J1H2	141.97 ± 9.09^d^
	−0.23	J1L1	147.10 ± 5.41^c^
	−0.15	F1H1	134.04 ± 10.43^ef^
	−0.88	F4H1	115.74 ± 8.37^g^
	−0.17	F4H2	167.10 ± 10.62^a^
	−0.08	M1H1	129.56 ± 6.42^f^
	+0.06	M1H2	143.63 ± 3.83^cd^
	−0.42	M1L2	136.01 ± 4.97^ef^
Medium	+0.12	F1L1	108.36 ± 3.77^hi^
	+0.11	F2H1	97.91 ± 3.70^k^
	−0.09	F3H1	110.55 ± 2.58^h^
	−0.05	F3H2	102.05 ± 5.49^jk^
	+0.07	F3L1	112.30 ± 5.06^gh^
	−0.47	M1L1	104.71 ± 7.99^ij^
Low	−0.43	F2L1	70.87 ± 6.92^n^
	+0.12	F3L2	69.27 ± 3.75^n^
	+0.02	F4L1	93.02 ± 6.11^l^
	+0.18	F4L2	41.09 ± 1.76^o^
	−0.04	M2L1	92.18 ± 3.05^l^
	−0.08	M2L2	75.97 ± 2.78^m^
	−0.08	M2L3	78.64 ± 9.91^m^

Superscript letters indicate statistically significant differences between groups (*p* < 0.05) as determined by multiple comparison tests. Different letters denote significantly different values.

Figure 2 presents heatmaps of Pearson correlation coefficients (r) among FAAs in raw laver (*Pyropia* spp.) samples classified into three quality grades (High, Medium, Low) based on TFAA content. Distinct compositional patterns were observed across the groups, directly linking amino acid correlations to quality classification. In the high-quality group, amino acids such as glutamic acid (Glu), aspartic acid (Asp), arginine (Arg), and valine (Val) exhibited moderate to strong positive correlations with other amino acids, with most correlations being positive. TFAA and alanine demonstrated a high correlation coefficient (r = 0.93), highlighting their potential as complementary indicators of raw laver quality. This consistency aligns with premium-grade classification criteria and supports the superior sensory and nutritional attributes associated with high-quality laver. The medium-quality group showed moderate correlations (e.g., Asp-Glu: r = 0.41) and transitional heterogeneity, with scattered positive correlations (r = 0.40–0.65) and near-zero values (r < 0.30). These patterns suggest variable amino acid accumulation, characteristic of intermediate-quality samples. In the Low-quality group, correlations were generally weak or negative (e.g., Glu-Ala: r = 0.28), reflecting compositional instability linked to inferior sensory properties and reduced market value, often the result of suboptimal postharvest handling. The heatmap analysis revealed that alanine consistently exhibited the highest correlation with TFAA across all quality grades, reinforcing its utility as a robust indicator of free amino acid content. Major umami-contributing amino acids, such as glutamic acid and aspartic acid, showed clearer correlation structures in the higher quality group. In contrast, the low-quality group showed disrupted amino acid correlations and compositional imbalance, interpreted as markers of quality degradation or biological stress.

The OPLS-DA score plot (Figure 3A) demonstrated strong model performance, with R^2^X = 0.790, R^2^Y = 0.916, and Q^2^ = 0.911, indicating excellent explanatory power, model fit, and predictive reliability. The three groups, High (green), Medium (blue), and Low (red), were clearly separated in the multivariate space. The High group clustered in the upper-right quadrant, whereas the Low group appeared in the lower-left quadrant, forming a gradient consistent with increasing TFAA content. Compared to the previous classification based on auction price (Figure 1), this model showed improved resolution and more distinct clustering. In the TFAA vector plot (Figure 3B), the vector pointed strongly toward the High group, illustrating that higher TFAA levels were the primary driver of this classification. The Medium group occupied an intermediate position between the High and Low groups, consistent with its moderate TFAA levels. The biplot (Figure 3C) revealed dense clustering of key amino acids (such as alanine, glutamic acid, and aspartic acid) near the High group, emphasizing their importance in defining high-quality profiles. These amino acids, which are known contributors to umami taste and nutritional value (36), were more abundant in high-TFAA samples. The loading contribution plot (Figure 3D) showed clear directional differences; variables associated with the High group had strong positive contributions, whereas those linked to the Low group showed negative contributions. Variables in the Medium group exhibited intermediate values, reinforcing their transitional classification. The VIP scores (Figure 3E) identified alanine (VIP = 1.3) and TFAA (VIP = 1.3) as the most influential discriminants, followed by total amino acids (VIP = 1.1). VIP values above 1.0 confirm their statistical significance and are consistent with prior findings on amino acid markers in laver quality (7). The distinct separation of the three TFAA-based groups supports the findings of Son and Lee (34), who reported amino acid-driven clustering in Korean seaweeds. The superior Q^2^ value of 0.911, compared to 0.85 reported by Ghallab et al. (37), further confirms the robustness of this TFAA-focused classification. This model offers a reliable and objective tool for evaluating raw laver quality, potentially replacing subjective, auction-based grading methods. The amino acid richness of high-TFAA samples supports their premium classification and market positioning. Future cultivation strategies could prioritize the enhancement of key amino acids, particularly alanine, to improve overall product quality. The OPLS-DA model effectively classified laver into three TFAA-based quality groups, with alanine and total FAAs emerging as the primary discriminative variables. The high Q^2^ value (0.911) of the model supports its potential application in industrial quality control and standardization.

### 3.4 Model validation through the confusion matrix

**Figure 4:**
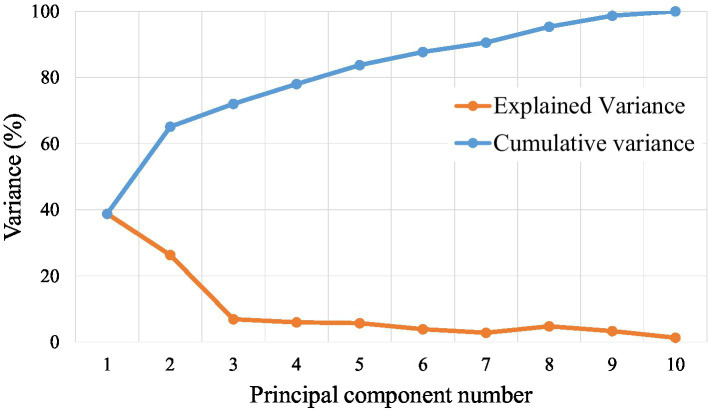
Explained variance ratio (red) and cumulative variance ratio (blue) for the first 10 principal components (PCs) used in the PLS-DA model.

**Figure 5:**
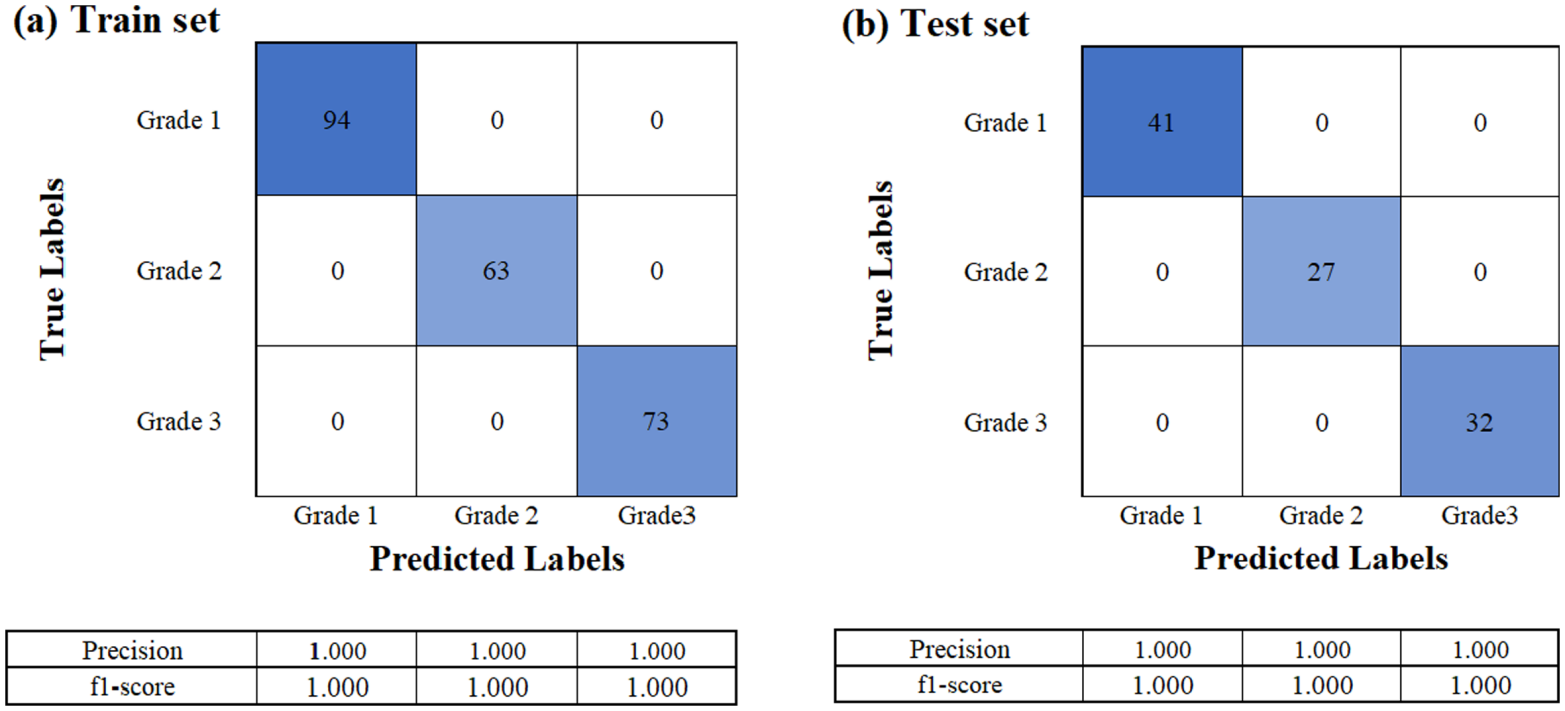
Confusion matrices of the PLS-DA classification model for raw laver grade prediction. **(a)** Confusion matrix for the training set (*n* = 230), **(b)** confusion matrix for the test set (*n* = 100).

The dataset consisting of a total of 330 raw laver samples was randomly divided into training and testing sets at a 7:3 ratio, resulting in 230 samples for training and 100 samples for testing. The response variable (Y data) used in the classification model was the grade of the raw laver, whereas the predictor variables (X data) included physicochemical properties such as moisture, ash, crude fat, and crude protein content. To determine the optimal number of PCs for the PLS-DA classification model, both the explained variance ratio and the cumulative explained variance for the first 10 PCs were calculated. The first seven PCs accounted for approximately 90.5% of the total variance, making them sufficient to retain most of the meaningful information from the original dataset (Figure 4). Therefore, the number of components was set to seven for model training. The classification performance of the PLS-DA model was evaluated using confusion matrices for both the training and test sets, as shown in Figure 5. In the training dataset (Figure 5A), the model achieved perfect classification, correctly predicting all 230 samples: 94 for Grade 1, 63 for Grade 2, and 73 for Grade 3. Similarly, in the test set (Figure 5B), all 100 samples were correctly classified without any misclassifications: 41 for Grade 1, 27 for Grade 2, and 32 for Grade 3. These results demonstrated 100% classification accuracy on both the training and test datasets, indicating excellent generalization performance and the absence of overfitting. The clear separation between classes confirmed the discriminative power of the selected physicochemical properties and the suitability of the PLS-DA model for predicting laver grades. Furthermore, the results suggest that the current grading system for raw laver is well-defined and effectively reflected its chemical composition, enabling reliable and objective classification through statistical modeling.

## 4 Conclusion

Laver (seaweed) has garnered global attention as a functional food ingredient, with South Korea emerging as a leading exporter of dried laver products. However, despite its economic significance, research on raw laver, the foundational material for all processed laver products, remains limited. This study addresses this critical gap by identifying scientifically validated quality markers for raw laver and providing data-driven insights to support transparent pricing in an otherwise opaque market. Additionally, the findings of this study underscore the importance of assessing raw laver quality prior to processing, as it provides the biochemical foundation for the final dried product. Our results can serve as a reference framework for future research aimed at bridging raw and dried laver quality, and specifically for evaluating whether nutritional and compositional indicators identified in raw laver can be transferred to dried laver classification systems. Such integration would enhance the industrial relevance of laver quality standardization and contribute to the further development of Korea’s laver industry.

Multivariate discriminant analysis based on TFAA content successfully classified raw laver (*Pyropia* spp.) into three distinct quality groups (High, Medium, Low) with high precision. The OPLS-DA model demonstrated exceptional performance, achieving R^2^X = 0.790, R^2^Y = 0.916, and Q^2^ = 0.911, confirming its robustness in explaining variance, fitting the data, and predicting group membership. The spatial separation of groups in the score plot (Figure 3A) followed a gradient aligned with TFAA content, confirming its effectiveness as a classification criterion. The key amino acids, glutamic acid, aspartic acid, and alanine, were identified as critical discriminants and clustered closely around the high group in the biplot (Figure 3C). These compounds, known for their roles in umami flavor and nutritional value, validated the premium status of high-TFAA samples. VIP scores further highlighted alanine (VIP = 1.3) and TFAA (VIP = 1.3) as primary drivers of group differentiation (Figure 3E), consistent with prior studies on seaweed biochemistry. The superiority of the model over traditional auction price-based classification (Figure 1) and its superior performance compared to similar studies [e.g., (37), Q^2^ = 0.85] underscore the advantages of using TFAA-focused variables. In addition, changes in the composition of laver are influenced by environmental factors. While no previous studies have directly examined the correlation between changes in TFAA content or amino acid profiles and environmental parameters, Park et al. (33) reported a strong negative correlation between sea surface temperature and crude protein content in laver (VIP = 2.00, r = −0.695, *p* < 0.05), as well as a significant correlation between heavy metal content and seawater salinity (VIP = 1.69, r = −0.667, *p* < 0.05). The observed relationship between protein content and sea surface temperature suggests that TFAA content may also be closely correlated with temperature changes. These findings further imply that TFAA could serve as a highly valid quality indicator for laver.

This approach bridges sensory evaluation with biochemical evidence, providing an objective framework for quality grading. Model validation using a confusion matrix confirmed the accuracy of the PLS-DA model. Using a 7:3 training–test split (230 training samples and 100 testing samples), the model achieved 100% classification accuracy in both datasets. The first seven PCs explained 90.5% of the total variance, ensuring sufficient information retention while avoiding overfitting. All 230 training samples were correctly classified (94 for Grade 1, 63 for Grade 2, and 73 for Grade 3), and all 100 test samples were accurately predicted (41 for Grade 1, 27 for Grade 2, and 32 for Grade 3). This exceptional generalization performance demonstrates the reliability and robustness of physicochemical property-based classification systems. Additionally, the findings suggest several practical implications: (1) industrial grading: the model identifies key compositional factors for objective quality evaluation of raw laver, providing scientific evidence to support traditional auction-based pricing; (2) market positioning: the biochemical richness of high-TFAA samples justifies their premium market value, aligning with consumer demand for nutrient-dense foods; (3) cultivation strategies: targeted enhancement of key amino acids (e.g., alanine or TFAA) may improve laver quality during cultivation and processing. Additionally, while our study offers critical insights into the biochemical quality indicators of raw laver based on samples from Seocheon collected between January and March, we recognize that this spatial and temporal confinement limits the generalizability of the findings. The Seocheon auction market mainly represents laver harvested from proximate aquaculture sites constrained by local tidal conditions, and the harvest season is shorter than in other regions such as Korea’s southern coasts. Consequently, the compositional profiles reported here may not fully capture interregional or seasonal variability present in laver production elsewhere in Korea or globally. To establish robust and broadly applicable quality standards, future research should include an expanded sampling strategy integrating multiple geographic locations, longer study durations, and potentially interannual monitoring. This would provide a comprehensive understanding of environmental and management influences on laver quality, thus reinforcing the industrial relevance and scalability of the proposed biochemical indicators.

Although the model demonstrated robust performance, its applicability across different geographic regions and seasonal conditions requires further validation. Future studies should incorporate multi-omics data (e.g., metabolomics, environmental factors) to enhance predictive accuracy and develop real-time monitoring systems. We are considering the integration of hyperspectral imaging technology to enable real-time monitoring and thereby enhance its practical applicability. Hyperspectral data for all collected samples have already been acquired, and modeling studies are currently in progress to establish predictive frameworks across different quality grades. We anticipate that this subsequent research will bridge the discovery of robust biochemical indicators with real-time, non-destructive analysis, ultimately enabling accurate classification of raw laver quality grades and enhancing its industrial value.

Additionally, to fully realize the industrial applicability of these findings, further validation across multiple cultivation regions, extended sampling over several years, and incorporation of comprehensive sensory panel evaluations are essential. Such expanded studies will enhance the robustness and generalizability of the proposed quality indicators, ultimately facilitating reliable and practical grading standards in the seaweed industry.

## Data Availability

The original contributions presented in the study are included in the article/Supplementary material, further inquiries can be directed to the corresponding authors.

## Supplementary material

SUPPLEMENTARY FIGURE 1 Map data ©2025 Google.

## Glossary

Proximate Composition: The analysis of the basic components of a sample, typically including moisture, protein, fat, and ash content. It helps in understanding the nutritional and physical properties of the sample.
Total Free Amino Acids (TFAA): A measure of the amino acids present in a sample that are not bound in proteins. These are often used as indicators of the nutritional value and flavor profile of foods.
Hyperspectral Imaging: A technique that captures and processes information across a wide range of wavelengths to analyze the composition and quality of materials, often used in food and agriculture industries for quality control.
Near-Infrared Spectroscopy (NIRS): A non-destructive analytical method used to determine the chemical composition of a sample by measuring its absorbance of near-infrared light. It’s commonly used for moisture and protein content analysis.
Attenuated Total Reflectance Fourier-Transform Infrared (ATR-FTIR) Spectroscopy: A technique that provides information about the molecular composition of a sample by measuring the absorbance of infrared light at different wavelengths. It’s widely used for surface analysis.
Confusion Matrix: A tool used in classification problems to evaluate the accuracy of a model by comparing the predicted labels against the actual labels. It summarizes the number of correct and incorrect predictions.
Kjeldahl Method: A widely used method for determining the nitrogen content in organic compounds, which is then multiplied by a conversion factor (6.25) to estimate the protein content.
Soxhlet Extraction: A method used to extract lipids (fats) from solid samples by continuously washing the sample with a solvent.

## References

[1] Pereira L. Edible seaweeds of the world. Boca Raton, FL, USA: CRC Press (2016).

[2] Wells ML Potin P Craigie JS Raven JA Merchant SS Helliwell KE. Algae as nutritional and functional food sources: revisiting our understanding. J Appl Phycol. (2017) 29:949–82. doi: 10.1007/s10811-016-0974-5, PMID: 28458464 PMC5387034

[3] El-Beltagi HS Mohamed AA Mohamed HI Ramadan KM Barqawi AA Mansour AT. Phytochemical and potential properties of seaweeds and their recent applications: a review. Mar Drugs. (2022) 20:342. doi: 10.3390/md20060342 35736145 PMC9227187

[4] Mišurcová L. (2011). Chemical composition of seaweeds. Handbook of marine macroalgae: Biotechnology and applied phycology (pp. 171–192).

[5] Nisizawa K Noda H Kikuchi R Watanabe T. The main seaweed foods in Japan. Hydrobiologia. (1987) 151-152:5–29. doi: 10.1007/BF00046102, PMID: 40956321

[6] Wen J Shi J Meng M Xu K Xu Y Ji D. Metabolic responses of Pyropia haitanensis to dehydration-rehydration cycles revealed by metabolomics. Mar Drugs. (2025) 23:203. doi: 10.3390/md23050203, PMID: 40422793 PMC12113544

[7] Ye Y Yang R Lou Y Chen J Yan X Tang H. Effects of food processing on the nutrient composition of *Pyropia* yezoensis products revealed by NMR-based metabolomic analysis. J Food Nutr Res. (2014) 2:749–56. doi: 10.12691/jfnr-2-10-15

[8] Ministry of Oceans and Fisheries. (2024). Annual report on Korean fisheries production. Republic of Korea: Ministry of Oceans and Fisheries

[9] Lee KI Lee GJ Yoon YS. A study on the use of FT-NIR spectophotometer for dried laver quality evaluation. J Marine Biosci Biotechnol. (2022) 14:69–75. Available at: http://koreascience.kr/article/JAKO202208660928324

[10] Derksen GC Blommaert L Bastiaens L Hasşerbetçi C Fremouw R van Groenigen J. ATR-FTIR spectroscopy combined with multivariate analysis as a rapid tool to infer the biochemical composition of Ulva laetevirens (Chlorophyta). Front Mar Sci. (2023) 10:1154461. doi: 10.3389/fmars.2023.1154461

[11] Jeong H Cho J Han J Yoon YS Kim HG Kim J. Large-area coverage–transmission near-infrared measurement of dried laver to determine crude protein content. Food Control. (2025) 168:110934. doi: 10.1016/j.foodcont.2024.110934

[12] Kim E Park JJ Lee G Cho JS Park SK Yun DY. Innovative strategies for protein content determination in dried laver (Porphyra spp.): evaluation of preprocessing methods and machine learning algorithms through short-wave infrared imaging. Food Chem. (2024) 23:101763. doi: 10.1016/j.fochx.2024.101763, PMID: 39286041 PMC11403402

[13] Lee JB Bae YJ Kwon GY Sohn SK Lee HR Kim HJ. Short-wave infrared hyperspectral image-based quality grading of dried laver (Pyropia spp.). Foods. (2025) 14:497. doi: 10.3390/foods14030497, PMID: 39942090 PMC11817384

[14] AOAC International. Official methods of analysis of AOAC international. (22nd ed.). New York, USA: Oxford University Press. (2023).

[15] Henderson JW Ricker RD Bidlingmeyer BA Woodward C. Rapid, accurate, sensitive, and reproducible HPLC analysis of amino acids. J Chromatogr B.(2000). 746, 85–104.

[16] Gómez-Alonso S Hermosín-Gutiérrez I García-Romero E. Simultaneous HPLC analysis of biogenic amines, amino acids, and ammonium ion as aminoenone derivatives in wine and beer samples. J Agric Food Chem. (2007) 55:608–13. doi: 10.1021/jf062820m, PMID: 17263449

[17] Eriksson L Byrne T Johansson E Trygg J Vikström C. Multi-and megavariate data analysis basic principles and applications, vol. 1. Umeå, Sweden: Umetrics academy (2013).

[18] Wold S Esbensen K Geladi P. Principal component analysis. Chemometr Intell Lab Syst. (1987) 2:37–52. doi: 10.1016/0169-7439(87)80084-9

[19] Galindo-Prieto B Eriksson L Trygg J. Variable influence on projection (VIP) for OPLS models and its applicability in multivariate time series analysis. Chemometr Intell Lab Syst. (2015) 146:297–304. doi: 10.1016/j.chemolab.2015.05.001

[20] Triba MN Le Moyec L Amathieu R Goossens C Bouchemal N Nahon P. PLS/OPLS models in metabolomics: the impact of permutation of dataset rows on the K-fold cross-validation quality parameters. Mol BioSyst. (2015) 11:13–9. doi: 10.1039/c4mb00414k, PMID: 25382277

[21] Chong I-G Jun C-H. Performance of some variable selection methods when multicollinearity is present. Chemometr Intell Lab Syst. (2005) 78:103–12. doi: 10.1016/j.chemolab.2004.12.011

[22] Huang BM Zha QL Chen TB Xiao SY Xie Y Luo P. Discovery of markers for discriminating the age of cultivated ginseng by using UHPLC-QTOF/MS coupled with OPLS-DA. Phytomedicine. (2018) 45:8–17. doi: 10.1016/j.phymed.2018.03.011, PMID: 29551643

[23] Worley B Powers R. Multivariate analysis in metabolomics. Curr Metabolomics. (2013) 1:92–107. doi: 10.2174/2213235X11301010092 26078916 PMC4465187

[24] Lasalvia M Capozzi V Perna G. A comparison of PCA-LDA and PLS-DA techniques for classification of vibrational spectra. Appl Sci. (2022) 12:5345. doi: 10.3390/app12115345

[25] Armstrong RA. When to use the Bonferroni correction. Ophthalmic Physiol Opt. (2014) 34:502–8. doi: 10.1111/opo.12131 24697967

[26] Duncan DB. Multiple range and multiple F tests. Biometrics. (1955) 11:1–42. doi: 10.2307/3001478

[27] Mok JS Lee TS Son KT Song KC Kwon JY Lee KJ. Proximate composition and mineral content of laver Porphyra yezoensis from the Korean coast. Korean J Fish Aquat Sci. (2011) 44:554–9. doi: 10.5657/KFAS.2011.0554

[28] Dawczynski C Schubert R Jahreis G. Amino acids, fatty acids, and dietary fibre in edible seaweed products. Food Chem. (2007) 103:891–9. doi: 10.1016/j.foodchem.2006.09.041

[29] Jung SM Kang SG Lee HJ Son JS Jeon JH Shin HW. Proximate composition and mineral content, amino acid of laver based on culture areas. Korean J Environ Ecol. (2016) 30:98–103. doi: 10.13047/KJEE.2016.30.1.098

[30] Kang MG Jeong MC Park SK Lee JW Cho JH Eom SH. Analysis of seasonal and regional changes in major food components of raw laver Pyropia sp. Korean. Fish Aquat Sci. (2018) 51:510–7. doi: 10.5657/KFAS.2018.0221

[31] Campos BM Ramalho E Marmelo I Noronha JP Malfeito-Ferreira M Mata P. Proximate composition, physicochemical and microbiological characterization of edible seaweeds available in the Portuguese market. Front Biosci. (2022) 14:26. doi: 10.31083/j.fbe1404026, PMID: 36575846

[32] Kusumo H. T. (1993) Chemical composition of Porphyra spp. in British Columbia, Canada. (Master’s thesis). British Columbia: Simon Fraser University, Department of Biological Sciences.

[33] Park SK Han SJ Jo DM Jo JB Lee G Park KJ. Influence of environmental factors on the proximate components and heavy metal contents of raw laver (Pyropia spp.) from South Korea. Mar Pollut Bull. (2025) 220:118372. doi: 10.1016/j.marpolbul.2025.118372 40633157

[34] Son SW Lee H. Comparative analysis of the amino acid composition and diversity of five seaweed species. Korean J Food Sci Technol. (2024) 56:123–32. Available at: https://koreascience.kr/article/JAKO202410280860981.page

[35] Guo YS Gong S Xie SM Chen AZ Jin HY Liu J. Mass spectrometry-based metabolomics investigation on two different seaweeds under arsenic exposure. Foods. (2024) 13:4055. doi: 10.3390/foods13244055, PMID: 39766997 PMC11675553

[36] Xiren GK Aminah A. Proximate composition and total amino acid composition of Kappaphycus alvarezii. Int Food Res J. (2017) 24:1255–60.

[37] Ghallab DS Shawky E Ibrahim RS Mohyeldin MM. Comprehensive metabolomics unveil the discriminatory metabolites of some Mediterranean Sea marine algae. Sci Rep. (2022) 12:7895. doi: 10.1038/s41598-022-12265-7 35577889 PMC9110716

