# Supplementary material for: Investigation of potential quality indicators for raw laver (Pyropia spp.) standardization: a collaborative approach between traditional assessment and analytical chemistry
Source: Front Nutr. 2025 Oct 1;12:1676911. doi: 10.3389/fnut.2025.1676911 (PMC12520933; doi:10.3389/fnut.2025.1676911)

Supplementary Table 1. Quantity, price, and related information for all raw laver samples auctioned on the sampling dates. Samples used in this study are indicated in bold and underlined.

| **No.** | **Jan. 30, 2024** | | **Feb. 6, 2024** | | **Feb. 13, 2024** | | **Feb. 20, 2024** | | **Feb. 26, 2024** | | **Mar. 6, 2024** | | **Mar. 13, 2024** | | |
| --- | --- | --- | --- | --- | --- | --- | --- | --- | --- | --- | --- | --- | --- | --- | --- |
|  | **Quantity** | **Price($)** | **Quantity** | **Price($)** | **Quantity** | **Price($)** | **Quantity** | **Price($)** | **Quantity** | **Price($)** | **Quantity** | **Price($)** | **Quantity** | **Price($)** |  |
| 1 | 80 | 183.46 | 110 | 123 | 90 | 131.15 | 32 | 145.92 | 43 | 175.54 | 80 | 221.31 | 96 | 224.62 |  |
| 2 | 80 | 185.54 | 89 | 127.08 | 80 | 128.85 | **30** | **140.69** | 60 | 188.85 | 80 | 222.46 | 75 | 229.62 |  |
| 3 | 90 | 186.54 | 51 | 127.85 | 49 | 111.69 | 60 | 152.15 | 40 | 175.23 | **75** | **193.08** | 50 | 237.54 |  |
| 4 | **80** | **182.99** | 80 | 127.08 | 85 | 120.15 | **80** | **145.54** | 70 | 182.00 | **70** | **146.08** | 27 | 154.77 |  |
| 5 | 39 | 171.69 | 59 | 127.08 | 100 | 152.15 | 80 | 152.69 | 70 | 184.62 | **27** | **153.08** | 32 | 223.00 |  |
| 6 | 60 | 178.85 | **60** | **119.62** | 100 | 142.69 | 50 | 156.85 | 50 | 189.69 | 50 | 231.62 | 110 | 247.31 |  |
| 7 | 60 | 178.38 | 90 | 130.77 | 70 | 94.62 | **55** | **160.15** | 50 | 174.31 | 50 | 226.92 | **40** | **211.62** |  |
| 8 | 90 | 177.69 | 100 | 135.23 | 83 | 144.62 | **15** | **165.38** | 60 | 176.92 | **60** | **210.23** | **51** | **212.38** |  |
| 9 | 70 | 174.23 | 70 | 129.92 | 70 | 117.69 | 50 | 150.00 | **29** | **78.46** |  |  | 50 | 211.62 |  |
| 10 | 46 | 170.77 | 77 | 128.15 | 100 | 116.38 | 33 | 150.46 | 70 | 192.31 |  |  | 66 | 223.92 |  |
| 11 | **71** | **173.23** | 120 | 131.69 | 40 | 93.08 |  |  | 70 | 192.31 |  |  | 70 | 219.23 |  |
| 12 | 70 | 162.31 | 110 | 128.62 | 17 | 76.92 |  |  | 70 | 190.62 |  |  | **65** | **216.23** |  |
| 13 | 70 | 158.92 | 70 | 133.08 | **89** | **137.31** |  |  | 17 | 189.69 |  |  | 19 | 221.15 |  |
| 14 | 60 | 183.62 | 90 | 135.08 | 68 | 129.92 |  |  | 80 | 193.08 |  |  | 38 | 217.85 |  |
| 15 | 60 | 177.69 | 80 | 138.46 | 50 | 142.77 |  |  | 80 | 183.77 |  |  | 88 | 232.69 |  |
| 16 | 60 | 157.69 | 32 | 133.54 | 72 | 111.54 |  |  | 70 | 176.85 |  |  | 110 | 204.62 |  |
| 17 | 55 | 171.62 | 60 | 136.92 | **70** | **73.23** |  |  | 70 | 152.69 |  |  |  |  |  |
| 18 | 55 | 173.08 | 50 | 133.15 | 70 | 106.15 |  |  | 80 | 200.85 |  |  |  |  |  |
| 19 | **50** | **146.92** | 80 | 133.23 | 69 | 134.77 |  |  | 58 | 197.38 |  |  |  |  |  |
| 20 | 70 | 170.23 | 110 | 142.46 | 67 | 137.62 |  |  | 40 | 189.08 |  |  |  |  |  |
| 21 |  |  | 71 | 139.38 | 91 | 137.62 |  |  | 35 | 188.92 |  |  |  |  |  |
| 22 |  |  | 90 | 143.46 | 80 | 129.85 |  |  | 79 | 188.62 |  |  |  |  |  |
| 23 |  |  | 70 | 142.77 | 72 | 144.23 |  |  | 40 | 183.77 |  |  |  |  |  |
| 24 |  |  | 70 | 142.77 | 84 | 127.08 |  |  | 45 | 196.62 |  |  |  |  |  |
| 25 |  |  | 70 | 143.31 | 100 | 114.46 |  |  | 6 | 184.62 |  |  |  |  |  |
| 26 |  |  | 71 | 143.54 | 110 | 114.54 |  |  | 42 | 191.46 |  |  |  |  |  |
| 27 |  |  | 50 | 146.23 |  |  |  |  | 110 | 191.62 |  |  |  |  |  |
| 28 |  |  | 70 | 147.62 |  |  |  |  | 72 | 191.46 |  |  |  |  |  |
| 29 |  |  | 100 | 146.08 |  |  |  |  | 79 | 171.62 |  |  |  |  |  |
| 30 |  |  | 70 | 145.15 |  |  |  |  | **70** | **186.15** |  |  |  |  |  |
| 31 |  |  | 70 | 145.31 |  |  |  |  | 70 | 178.54 |  |  |  |  |  |
| 32 |  |  | 68 | 147.92 |  |  |  |  | **36** | **163.85** |  |  |  |  |  |
| 33 |  |  | 80 | 148.54 |  |  |  |  | **74** | **205.31** |  |  |  |  |  |
| 34 |  |  | 77 | 149.85 |  |  |  |  |  |  |  |  |  |  |  |
| 35 |  |  | 80 | 153.85 |  |  |  |  |  |  |  |  |  |  |  |
| 36 |  |  | **70** | **151.46** |  |  |  |  |  |  |  |  |  |  |  |

Supplementary figure 1.


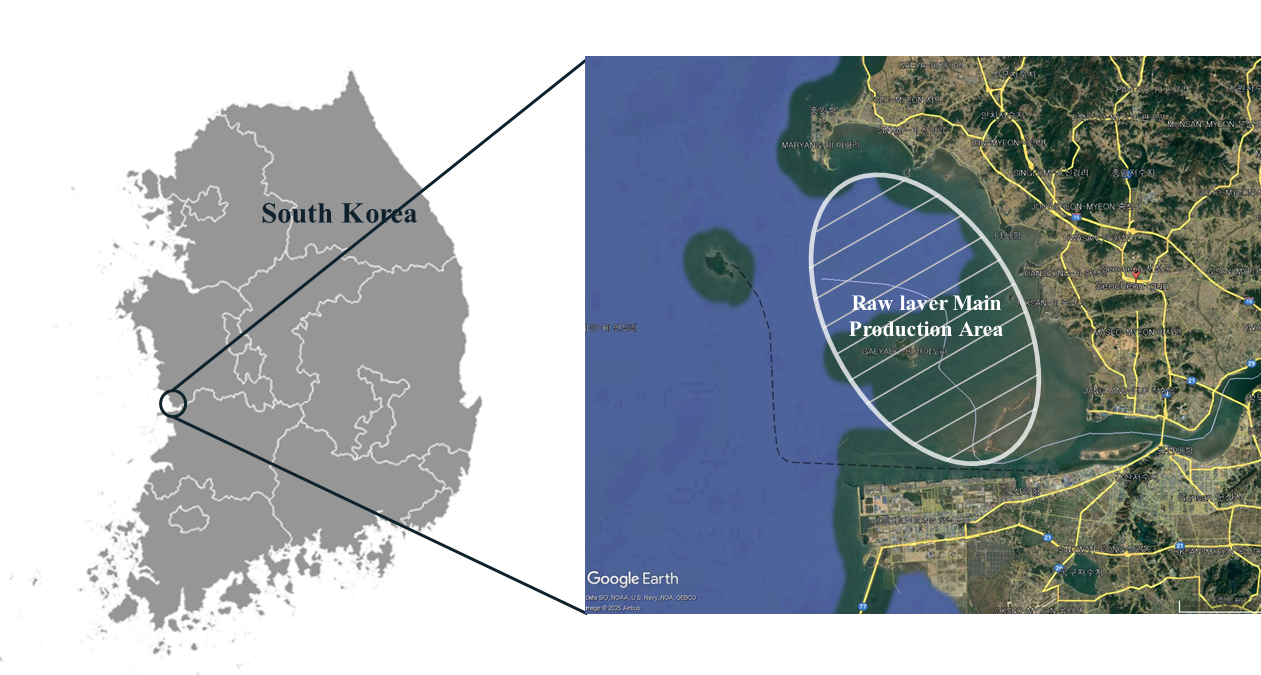

Supplement: Supplementary file 1 [file Table_1.docx]
